# Frequency of five unusual presentations in patients with COVID-19: results of the UMC-19-S_1_

**DOI:** 10.1017/S0950268820001910

**Published:** 2020-08-26

**Authors:** Òscar Miró, Pere Llorens, Sònia Jiménez, Pascual Piñera, Guillermo Burillo-Putze, Alfonso Martín, Francisco Javier Martín-Sánchez, Juan González del Castillo

**Affiliations:** 1 Emergency Department, Hospital Clínic, IDIBAPS, University of Barcelona, Barcelona, Catalonia, Spain; 2 Emergency Department, Hospital General de Alicante, University Miguel Hernández, Elche, Alicante, Spain; 3 Emergency Department, Hospital Reina Sofía, Murcia, Spain; 4 Emergency Department, Hospital Universitario de Canarias, Tenerife, Spain; 5 Emergency Department, Hospital Severo Ochoa, Leganés, Madrid, Spain; 6 Emergency Department, Hospital Clínico San Carlos, IDISSC, Univesdad Complutense, Madrid, Spain

**Keywords:** COVID, Guillain–Barré syndrome, meningoencephalitis, myopericarditis, pancreatitis, SARS-CoV-2, spontaneous pneumothorax

## Abstract

Despite SARS-CoV-19 infection has a stereotypical clinical picture, isolated cases with unusual manifestations have been reported, some of them being well-known to be triggered by viral infections. However, the real frequency in COVID-19 is unknown. Analysing data of 63 822 COVID patients attending 50 Spanish emergency department (ED) during the COVID outbreak, before hospitalisation, we report frequencies of (myo)pericarditis (0.71‰), meningoencephalitis (0.25‰), Guillain–Barré syndrome (0.13‰), acute pancreatitis (0.71‰) and spontaneous pneumothorax (0.57‰). Compared with general ED population, COVID patients developed more frequently Guillain–Barré syndrome (odds ratio (OR) 4.55, 95% confidence interval (CI) 2.09–9.90), spontaneous pneumothorax (OR 1.98, 95% CI 1.40–2.79) and (myo)pericarditis (OR 1.45, 95% CI 1.07–1.97), but less frequently pancreatitis (OR 0.44, 95% CI 0.33–0.60).

## Introduction

The clinical picture of infection by SARS-CoV-2 is quite stereotypical, being mainly characterised by fever and respiratory symptoms, with dyspnoea and lung infiltrates in the most severe cases [1]. However, isolated case reports and short case series have communicated unusual clinical manifestations in patients with COVID-19, although it is not known if these cases are merely coincidental as the result of complications of patient management or treatments provided during hospitalisation or, alternatively, SARS-CoV-2 predisposes to these manifestations. Particularly, this is reportedly the case with acute (myo)pericarditis [2, 3], meningoencephalitis [4], Guillain–Barré syndrome [5], acute pancreatitis [6] or spontaneous pneumothorax [7]. As these processes have been described as a direct-effect or an immunologically-mediated consequence of infection of viruses other than SARS-CoV-2 [8–10], it would be plausible that SARS-CoV-2 could also favour such entities. Focusing on patients with COVID-19 at emergency department (ED) arrival, before hospitalisation and the initiation of specific drugs for SARS-COV-2 infection could help to establish the real frequency of these unusual manifestations. Effectively, this approach would allow estimating the relative frequencies in COVID patients with respect to general ED population and, in the end, interpret whether they are or are not connected with the pathogenesis of SARS-CoV-2. Accordingly, the main objective of the present study was to estimate the relative frequencies for the five abovementioned manifestations (acute (myo)pericarditis, meningoencephalitis, Guillain–Barré syndrome, acute pancreatitis and spontaneous pneumothorax) in COVID patients coming to the ED during the COVID-19 outbreak and compare them with the relative frequencies observed in non-COVID ED comers. To achieve this objective, we carried out the present study in 50 Spanish EDs included in the SIESTA (Spanish Investigators in Emergency Medicine TeAm) research network.

## Methods

### Study design and setting

The present study forms part of the Unusual Manifestations of COVID-19 (UMC-19) project, which was designed to investigate the potential relationship between COVID-19 and 10 different entities that could be influenced by SARS-CoV-2 infection itself. The UMC-19 project was designed as a retrospective, multicentre study carried out in Spanish EDs integrated in the SIESTA research network. Detailed description of the full project has been published elsewhere [11, 12].

As part of the UMC-19 project, the present study (study 1, UMC-19-S_1_) was designed to determine the individual relative frequency of each of the five following entities in COVID patients (all corresponding to diseases that have been reported to be associated with viral infections, and for which at least one case had been published in patients with COVID-19): acute (myo)pericarditis, acute meningoencephalitis, Guillain–Barré syndrome, acute pancreatitis and spontaneous pneumothorax. We accordingly included all COVID patients attending the 50 EDs participating in the present study (17% of the EDs of the Spanish public health system) that were diagnosed with one of the five aforementioned entities. For these patients, we reviewed the diagnosis of COVID-19 and we accepted both, those cases with a COVID diagnosis based on detection of the SARS-CoV-2 RNA in nasal-pharyngeal swab using reverse transcriptase polymerase chain reaction (RT-PCR) tests and those cases in whom, due to the shortage of SARS-CoV-2 tests during the pandemic outbreak, diagnosis was exclusively based on clinical criteria. To accept the clinical diagnosis of COVID-19, we required that patient complained with the typical COVID-19 symptoms (including fever, cough, dyspnoea, dysgeusia and anosmia) and radiological abnormalities associated with COVID-19 (bilateral lung interstitial infiltrates and/or glass-ground patchy pattern). When available, same findings in lung CT were also considered. Since the first case of SARS-Cov-2 infection was detected in Spain on 31 January 2020, we defined the COVID period for inclusion of COVID patients 1 March to 30 April 2020 (2-month period).

On the other hand, during this 2-month COVID period, we identified all non-COVID patients coming to the ED and being diagnosed with one of the five entities under investigation previously mentioned. In addition, we also identified all patients with the same diseases diagnosed in the ED during the same 2-month period (from 1 March to 30 April, 2-month period) of 2019 (non-COVID period, all patients being non-COVID).

The identification of COVID and non-COVID patients with each of these five entities was first carried out through an electronic system according to specific codes and, thereafter, medical reports of these patients were manually reviewed and confirmed by the principal investigator of each centre according to definitions accepted by scientific societies. Only diagnoses performed in the ED, before patient hospitalisation, were considered, while patients developing such manifestations along hospitalisation were specifically excluded from the analysis.

Finally, in order to obtain the relative frequencies for every particular entity in each group of patients (COVID and non-COVID), the total number of patients attending the ED during each period were recorded. For the 2020 period, we distinguished between the number of patients with a diagnosis of COVID-19 (clinical and/or microbiological) and the rest of the patients (non-COVID). For the 2019 period, all ED comers were considered non-COVID.

### Ethics

The UMC-19 project was approved by the Ethics Committee of the Hospital Clínic of Barcelona (Spain), (acting as the central ethical committee) with the reference number HCB/2020/0534. UMC-19-S1 was carried out in strict compliance with the principles of the Declaration of Helsinki.

### Statistical analysis

Relative frequencies for each entity were expressed per thousand (‰) COVID or non-COVID patients coming to the ED with 95% confidence interval (CI). Differences of relative frequencies between COVID and non-COVID populations were expressed as unadjusted odds ratios (ORs) with 95% CI. We calculated all the OR for COVID patients using all the non-COVID patients together as reference, but also recalculating OR using the non-COVID patients included in the non-COVID period and COVID period separately. In all comparisons, statistical significance was accepted if the 95% CI of the risk estimations excluded the value 1. The analyses were performed with the SPSS version 24 (IBM, Armonk, New York, USA).

## Results

During the 2-month period of COVID-19 outbreak, 63 822 COVID patients were diagnosed in the 50 Spanish EDs participating in the UMC-19-S_1_. In this group, we identified 45 acute (myo)pericarditis (relative frequency: 0.71‰, 95% CI 0.51–0.94), 16 acute meningoencephalitis (0.25‰, 0.14–0.41), 8 Guillain–Barré syndrome (0.13‰, 0.05–0.25), 45 acute pancreatitis (0.71‰, 0.51–0.94) and 36 spontaneous pneumothorax (0.57‰, 0.40–0.78). Of these 150 patients, SARS-CoV-2 infection was confirmed in by RT-PCR in 98 cases (65.3%), while in the remaining 52 cases COVID-19 diagnosis was clinical.

On the other hand, we reviewed the diagnosis of 1 125 491 non-COVID patients (782 125 included in the pre-COVID period; 343 366 included in the COVID period), and we identified 546 acute (myo)pericarditis (0.49‰, 0.45–0.53), 181 acute meningoencephalitis (0.16‰, 0.14–0.19), 31 Guillain–Barré syndrome (0.03‰, 0.02–0.04), 1787 acute pancreatitis (1.59‰, 1.52–1.66) and 321 spontaneous pneumothorax (0.29‰, 0.26–0.32).

**Fig. 1.**
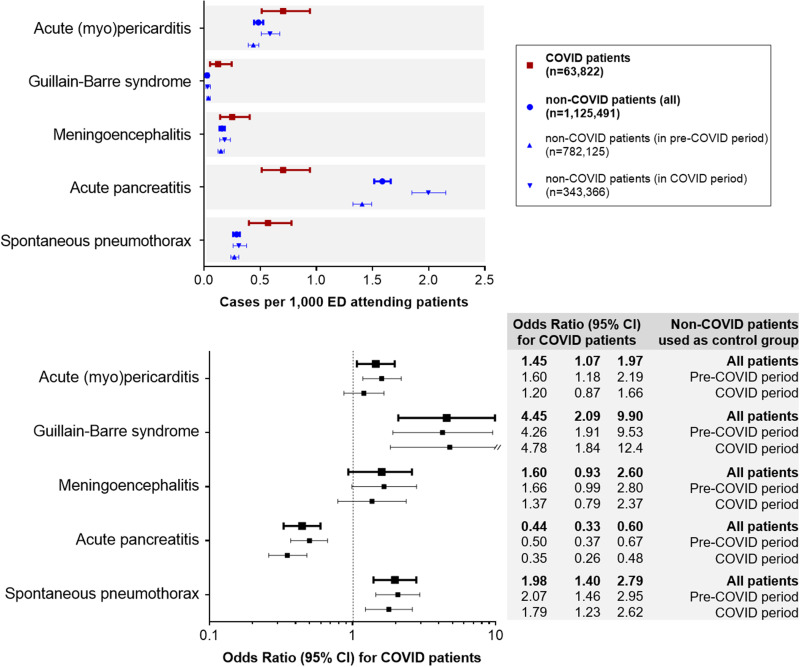
Comparison of the incidence of the five unusual manifestations evaluated in patients with COVID-19 compared with non-COVID patients.

Based on these relative frequencies, the Guillain–Barré syndrome (OR 4.55, 95% CI 2.09–9.90), spontaneous pneumothorax (1.98, 1.40–2.79) and acute (myo)pericarditis (1.45, 1.07–1.97) were significantly more frequent in COVID than in non-COVID patients in the ED. On the other hand, acute pancreatitis was less frequent (0.44, 0.33–0.60). We did not find statistically significant differences in acute meningoencephalitis (1.60, 0.93–2.60). Similar results were found in all OR when comparing COVID patients with non-COVID patients recruited during the pre-COVID and the COVID periods separately (Fig. 1).

## Discussion

We quantified the frequency of five manifestations that could potentially be directly produced by the patient's inflammatory response or immunologic stimulation as consequence of SARS-CoV-2 infection. They were measured at the time of patient ED consultation, before hospitalisation. We acknowledge that our figures do not correspond to the real relative frequency of these entities in the COVID-19 pandemic. On the one hand, only about 10% of COVID patients consulted the ED during the pandemic in Spain, therefore, the incidences could be up to 10 times lower. On the other hand, manifestations developed during hospitalisation were not taken into account, and their inclusion could have led to an (unknown) increase of our estimates. Finally, although it is foreseeable that the vast majority of patients presenting one of these five entities will consult to ED due to related signs or symptoms, asymptomatic or minor forms could also have remained undetected. However, to the best of our knowledge, this is the first reliable estimation of the relative frequencies of these five manifestations, which were very low, ranging from 0.13 cases per thousand patients attending the ED for Guillain–Barré syndrome to 0.71 cases for acute pancreatitis.

The second notable finding is that Guillain–Barré syndrome, spontaneous pneumothorax and acute myocarditis were more frequently observed in COVID patients than in non-COVID patients in the ED. We acknowledge that comparison of COVID patients with the rest of ED comers altogether could be unfair to demonstrate whether these frequencies are really higher than expected. However, if we accept that the vast majority of patients with Guillain–Barré syndrome, spontaneous pneumothorax and acute (myo)pericarditis attend the ED for symptom consultation, then our data suggest that there is, indeed, an increased risk in COVID patients. Remarkably, using the same comparator for all five processes (non-COVID patients in the ED, altogether or separated by pre-COVID and COVID period ED comers), what seems clear is that Guillain–Barré syndrome is increased more than fourfold while increases were lower for the remaining processes, or were even decreased (acute pancreatitis). In fact, despite Guillain–Barré syndrome being the most unusual of the five manifestations evaluated in the UMC-19-S_1_, a very recent review identified at least 10 cases reported in COVID patients [5]. Taking all of this into account, we believe our data confirm and expand the suspicion of the causative role of SARS-CoV-2 in this syndrome, and it should be added to the list of viral infections able to promote an aberrant autoimmune response with cross-reaction against gangliosid components of the peripheral nerves including cytomegalovirus, Epstein-Barr virus, influenza-A virus, hepatitis E and Zika virus [8]. With respect to the previously known coronaviruses, we have found no reported cases of Guillain–Barré related to SARS-CoV outbreak, while for the MERS-CoV it has been recently two patients developing Guillain–Barré syndrome in South Korea [13]. However, as outbreaks of SARS-CoV and MERS-CoV have been very limited geographically and the whole number of infected patients worldwide remains low, it is still unknown the real incidence of Guillain–Barré syndrome for these coronaviruses.

We believe our findings contribute to a better knowledge of COVID-19, especially from an epidemiological perspective. On the one hand, they suggest a link between SARS-CoV-2 infection and pathogenesis of some unusual manifestations that had been reported just as isolated cases until now: Guillain–Barré syndrome, acute (myo)pericarditis and spontaneous pneumothorax. The exact mechanisms involved in such pathogenesis, as well as the full description of the clinical picture in patients with COVID-19 will have to be defined by future studies. Conversely, other manifestations that have been suggested to be increased in COVID-19 patients, as acute pancreatitis, are not supported by our findings. Finally, once the COVID-19 outbreak ends, the SARS-CoV-2 infection must not be forgotten in every patient diagnosed with the conditions we have found to be increased in COVID patients (i.e. Guillain–Barré syndrome, acute (myo)pericarditis and spontaneous pneumothorax). Accordingly, detection of SARS-CoV-2 should be performed in order to determine aetiology of cases initially considered as idiopathic.

Some limitations impose caution in interpreting our findings. First, patient-related or disease-related factors could, to some extent, have accounted for decreased/increased ORs, and we did not adjust for them. Second, ED patients and disease typology could have differed during the COVID outbreak (due to country lockdown), although the similar rates of the five manifestations observed for COVID patients using both subgroups of controls (from the pre-COVID and the COVID periods) do not support this possibility. Third, in one-third of cases with unusual manifestations, the diagnosis of SARS-CoV-2 infection was based on clinical/radiological findings with no microbiological confirmation. Fourth, the COVID-19 pandemic, emergency physicians have had a lower threshold for ordering some diagnostic studies, and some diagnoses could have been increased. Fifth, as the number of patients with unusual manifestations was low, a type-II error could be present in our estimations. As an example, the estimation of 60% of increase in relative frequency of meningoencephalitis in COVID patients respect to non-COVID patients found in the present study resulted statistically non-significant. Sixth, the overall increase of risk in COVID patients for some manifestations could have been even higher if complications developed during admission had been taken into account. This could be the case of spontaneous pneumothorax, which it has been reported to be increased in mechanically ventilated suffering from lung inflammatory processes in general, and from pulmonary infections in particular [11, 12]. Seventh, we considered the whole ED census (as in many EDs participating in the study such a census does not contain disaggregate data) to obtain the number of COVID and non-COVID patients during the studied periods. This included accident/violence cases, which correspond to non-COVID patients in the vast majority. Accordingly, risk in COVID patients could have resulted slightly overestimated due to this fact.

In conclusion, our results suggest a clear association of SARS-CoV-2 infection with Guillain–Barré syndrome and, to a lesser extent, with (myo)pericarditis. The underlying mechanisms leading to this increased risk should be investigated. Remarkably, once the COVID-19 outbreak ends, the SARS-CoV-2 infection must not be forgotten in every patient diagnosed with the conditions we have found to be increased in COVID patients (i.e. Guillain–Barré syndrome and acute (myo)pericarditis and spontaneous pneumothorax) and, accordingly, SARS-CoV-2 infection should be systematically investigated.

## Data Availability

The data that support the findings of this study are available from Dr Òscar Miró and Dr Juan González del Castillo. Any request for review and use will have to be addressed to them, and will need the approval of the steering committee of the SIESTA Research group.
